# 3D characterization of low optical absorption structures in large crystalline sapphire substrates for gravitational wave detectors

**DOI:** 10.1038/s41598-020-80313-1

**Published:** 2021-01-29

**Authors:** Manuel Marchiò, Matteo Leonardi, Marco Bazzan, Raffaele Flaminio

**Affiliations:** 1 Department of Astronomy, The University of Tokyo, Tokyo, 113-8654 Japan; 2 Gravitational Wave Science Project, National Astronomical Observatory of Japan, Mitaka, Tokyo, 181-0015 Japan; 3 Dipartimento di Fisica e Astronomia, Università di Padova, Padua, 35131 Italy; 4 Laboratoire d’Annecy de Physique des Particules (LAPP), Université Grenoble Alpes, Université Savoie Mont Blanc, CNRS/IN2P3, 74941 Annecy, France

**Keywords:** Astronomical instrumentation, Materials for optics, Optical materials and structures

## Abstract

Very high-quality sapphire substrates are key elements of the cryogenic Japanese gravitational interferometer KAGRA, in which they are used to build the main mirrors, working as the test masses to sense the gravitational waves. To meet the extreme requirements of this system, the sapphire test masses must possess an extremely low optical absorption, which makes their study challenging using standard methods. In this paper, we illustrate the results obtained on two typical samples using a specialized absorption setup based on the technique of Photo-thermal Common-path Interferometry (PCI). Our system combines a very high sensitivity to small absorption features with the possibility to perform a full three-dimensional mapping of the sample volume. Our results elucidate how the ultra-low absorption variations inside the sample possess a structure that is probably inherited from the growth history of the sample. Some conclusions on the role of structural defects as preferential sites for the inclusion of absorbing centers are drawn.

## Introduction

Laser interferometers such as Advanced LIGO^1^, Advanced Virgo^2^ and KAGRA^3^ are currently the most sensitive instruments to detect gravitational waves (GW), being able to sense interferometer arm length changes as small as ${\sim } 1 \times 10^{-21} \, \hbox {m}$ at a frequency of $100\, \hbox {Hz}$. In order not to spoil such a delicate measurement, most of the research effort consists in improving the Signal-to-Noise Ratio of the interferometer output by reducing unwanted noise sources such as seismic, thermal, and quantum noises^4^.

The Japanese detector KAGRA is the first GW detector to operate at cryogenic temperature to reduce thermal noise. At the design temperature of $20\, \hbox {K}$ usual substrate materials, such as amorphous silica, are not a viable solution because of their low thermal conductivity as well as high mechanical losses at low temperatures^5^. Instead, KAGRA test masses are designed to be made of crystalline sapphire. Sapphire ($\mathrm {\alpha -Al_{2}O_{3}}$) is a well-known crystalline oxide material with trigonal symmetry belonging to the $R{\overline{3}}c$ space group. Its wide transparency range from UV to $5000\, \hbox {nm}$ and its high hardness coupled with a high thermal conductivity of around $3000\, \hbox {W} \, {\hbox {m}}^{-1} \, {\hbox {K}}^{-1}$ and a low thermo-optic coefficient at $20\, \hbox {K}$ temperature^6^ make it an excellent candidate. However, the presence of absorbing impurities such as color centers or other point defects constitutes a serious issue. In modern GW interferometers, the sensitivity is improved by keeping the amount of circulating optical power as high as technically possible (order of hundreds of $\hbox {kW}$). Therefore, even a small optical absorption in the mirrors can generate a significant amount of heat to be dissipated by conduction through the suspension fibers, which are also made of sapphire. However, the suspension thermal noise increases with the fiber diameter, so the latter must be kept as small as possible. Therefore, the maximum amount of heat that can be extracted is limited by the small section of the suspension fibers. In the KAGRA design^7^, the suspension cryostat is designed to extract $1.2\, \hbox {W}$ from each test mass. According to Lambert-Beer’s law, the fraction of absorbed power from the laser is $[1-e^{-\alpha L}]$, where $\alpha$ is the absorption coefficient and *L* is the thickness of the absorbing medium. This datum, combined with the thermal conductivity of the fibers, sets the requirement of less than $1 \, \hbox {ppm}$ of total surface absorption from the coating, and less than $50 \, \hbox {ppm/cm}$ for the absorption coefficient of the bulk sapphire substrate.

The fabrication of large size sapphire substrates (${23} \, \hbox {kg}$ in the case of KAGRA) with such low absorption at $1064 \, \hbox {nm}$ is a technological challenge for crystal growing companies. The causes of sapphire absorption are not completely understood; they may depend on the growth procedure and on the material purity. Moreover it is important for the final application in KAGRA to discriminate between samples having different absorption structures. A high sensitivity and spatially resolved sample-by-sample characterization of the sapphire substrates absorption is therefore mandatory. In response of this need, we set up an experiment to measure the absorption of large substrates prepared for KAGRA with 3D resolution. In the Experiment section we explain the details of the experiment that we set up to measure KAGRA mirrors. In the Results section we analyze the result of the measurement, and in the last section we draw the conclusions of this work.

## Experiment

### Experimental set-up

The system is based on the so-called Photo-thermal Common-path Interferometry (PCI) method^8^. This is a pump - probe technique that exploits the thermo-optic effect (i.e. the change in the material’s refractive index upon a temperature increase) to visualize weak absorption features with a sub-mm spatial resolution.

**Figure 1:**
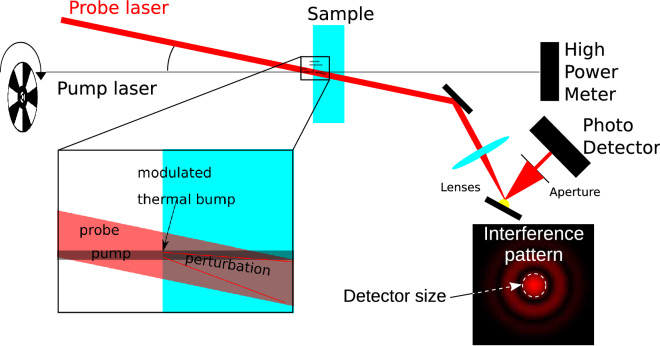
Concept diagram of the Photo-thermal Common-path Interferometer (PCI) method. The pump laser is a 20 W continuous-wave fiber-amplified diode laser at 1064 nm. The probe laser is a low-noise 5 mW Helium-Neon laser. The imaging unit (made by a converging lens) magnifies the interference pattern on the photo-detector. As the size of the photo-detector is smaller than the probe spot the intensity variation of the central part of the beam results in a periodic variation of the power detected by the sensor.

In Fig. 1 a conceptual scheme of the setup is depicted. A high-power laser beam produced by a continuous wave fiber-amplified diode laser at $1064 \, \hbox {nm}$ (hereafter referred to as *pump*) is focused inside the sample which is scanned with the aid of a computer-controlled translation stage. The focused beam creates a local increase of the sample temperature, which in turn modifies the local refractive index of the material. The pump is modulated by an optical chopper at a fixed frequency so that the temperature change is modulated as well. As the power absorbed by our sample is very low, the amplitude of the temperature modulation can be taken to be proportional to the absorbed power and thus to the local absorption coefficient. Due to thermo-optical effects, a periodic modulation of the refractive index is thus created along the pump beam path:

1 $$\begin{aligned} \Delta n(t)\propto \alpha P(t)\frac{dn}{dT} \end{aligned}$$

where $\alpha$ is the absorption coefficient, *P*(*t*) is the periodically modulated pump power and *dn*/*dT* is the thermo-optic coefficient. It should be noted that this latter term may include also secondary contributions such as for example thermal strain-mediated refractive index changes. This is not posing particular problems to our treatment as long as the refractive index change can be considered proportional to the temperature change.

This effect can be sensed by a low-intensity *probe* beam, that crosses the pump beam at a small angle, produced by a low-noise 5 mW Helium-Neon laser. In fact, the refractive index gradient induces a distortion of the probe beam wavefront. After a free space propagation, this results in a time-modulated diffraction pattern. Since the pattern modulation is of purely refractive nature, the overall power of the probe is conserved. However the active area of the photodiode includes only the central portion of the probe spot, the time-varying intensity pattern is converted into a modulated electrical signal at the same frequency of the pump modulation. Again, to the first order, we can safely assume that this AC amplitude is proportional to the phase modulation and thus to the local absorption coefficient. The overall proportionality constant is determined empirically by measuring a sample of known absorption, as detailed in the next section. The two beams cross at the waist position and the resolution limit along the X and Y directions is given by the pump beam diameter which is $72\, \upmu \hbox {m}$, while the probe is about 3 times larger. The Z resolution instead is given by the length of the interaction volume between pump and probe. Given the angle between the two beams ($0.1\, \hbox {rad}$ outside the sample), the length of the interaction volume along the Z direction is 2 mm in the case of a sapphire sample.

The sample sits on a large translation stage that moves along the three directions X, Y, and Z. As the absorption is measured at the crossing point between pump and probe, an absorption map can be obtained with a resolution given by the interaction volume, as detailed above. The whole system is controlled by a LabVIEW program that moves the translation stages and acquires the data.

### Data analysis and calibration procedure

The absorption coefficient $\alpha \, [\text {cm}^{-1}]$ is calculated using the following formula:

2 $$\begin{aligned} \alpha =\frac{V_p}{V_\text {Ave}\cdot P\cdot R} \end{aligned}$$

where $V_p$ is the AC amplitude of the probe signal, $V_\text {Ave}$ is the DC component, *P* is the pump power, and $R \, [\text {cm}/\text {W}]$ is the calibration factor which is determined by measuring a sample with known absorption. The sensitivity of the system is maximized when the detector is placed at one Rayleigh distance from the interaction volume^8^. The distortion of the probe intensity profile can be seen as the far-field diffraction pattern of the refractive index perturbation. A telescope system therefore is used to map this point on the detector position.

Because of the sample refraction, the probe beam exiting from the back sample surface is displaced of an amount that depends on the sample thickness and that may be easily derived from the Snell’s law:

3 $$\begin{aligned} d_\text {shift}=\frac{n-1}{n}L \end{aligned}$$

where *n* is the refractive index of the test sample, and *L* is the thickness of the test sample. In order to allow for different thicknesses, the imaging unit is mounted on a micrometric translation stage allowing us to re-center it along the final beam path.

Furthermore, due to refraction, when scanning the sample from one surface to the other, the thickness appears to be $\sim 1.8$ times thinner. In particular, to scan a 150 mm thick sample the translation stages only moves by 85 mm. Therefore, in the plots shown in this article, the Z coordinate has been scaled to match the physical position inside the substrate. The sensitivity of the setup for a sapphire sample is as good as 1.5 ppm/cm. This is the smallest absorption that can be measured. The accuracy is about 20%. The optical power absolute measurements that are needed for the calibration are the main limit to accuracy^8^.

### Samples

In this work, we report the results from two sapphire samples obtained from a boule grown by a modified Kyropoulos method in which, after melting the raw material in a crucible, an oriented seed is put in contact with the bath and the temperature is decreased in a controlled way so that the crystal grows into the melt. The seed is slowly pulled and rotated, only to control the growth process (rather than to establish a mass transport as in the Czochralski technique). The pulling direction corresponds to the *c*-axis of the crystal.

The first sample (Sample A) is a 23 kg polished sapphire optical substrate made for KAGRA extracted from the center of the boule. The size is 220 mm in diameter and 150 mm in thickness. The crystal is oriented with the *c*-axis along the optical axis of the mirror in order to minimize birefringence effects. To characterize Sample A, we mapped circular areas on the XY plane at three different depths in the sample: at about 1 cm from the first surface; in the center; and at about 1 cm from the second surface. The maps are 120 mm in diameter and 1 mm of resolution. Then we mapped two rectangular areas, one on the XZ plane and one on the YZ plane that cross the central axis of the sample. The size is 120 mm along X and Y (the same as the circular maps), and 105 mm along Z (enough to scan the sample from one surface to the other). The resolution is 1 mm. Since every point absorption takes about 1 s to be measured, each map takes approximately 9 hours to be completed. Given the beam diameter on KAGRA’s mirror of about 70mm, the chosen map size ensures a coverage of 99.7% of the beam power. This represent a good trade-off between beam size coverage and time required for each map.

The second sample (Sample B) is a smaller sample having size 50.8 mm in diameter and 20 mm in thickness with the optical axis aligned along the *c*-axis. This sample was carved from a peripheral part of the boule. In this case, we acquired a map on the XY plane with a 1 mm resolution plus a higher resolution map of the central area with a stepsize of 0.05 mm in both X and Y direction. We also acquired a lateral map on the XZ plane. Thanks to the smaller size of this sample, after the optical absorption measurement it was possible to mount it into a Philips MRD X-ray diffractometer equipped with an Eulerian cradle, in order to relate the observed absorption features with the crystallographic directions.

## Results

**Figure 2:**
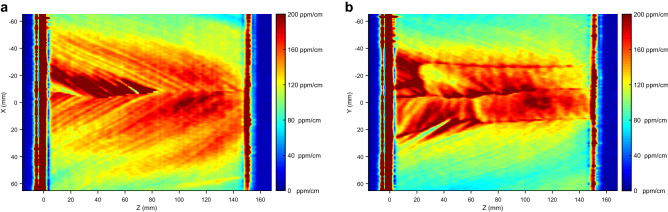
Rectangular absorption maps of the sapphire substrate on the XZ plane **(a)** and YZ plane **(b)**. The dark blue areas correspond to the 0 absorption outside the sample. On the surfaces, there is a large absorption that saturates on the colorbar.

In Fig. 2a,b the rectangular maps for Sample A are shown. Those maps show the presence of absorption stripes periodically repeating along the growth direction of the sample with a step of roughly $7\, \hbox {mm}$, which is close to the resolution limit along z of our system ($\simeq 2\, \hbox {mm}$), but still measurable. The highly absorbing stripes in Fig. 2a appear to be inclined of about 30 degrees with respect to the *c*-axis suggesting that those absorption features are related to the growth interface of the boule. The situation is less obvious in Fig. 2b, where, although some features remind the presence of stripes, we rather observe some highly absorbing lines oriented nearly parallel to the *c*-axis.

**Figure 3:**
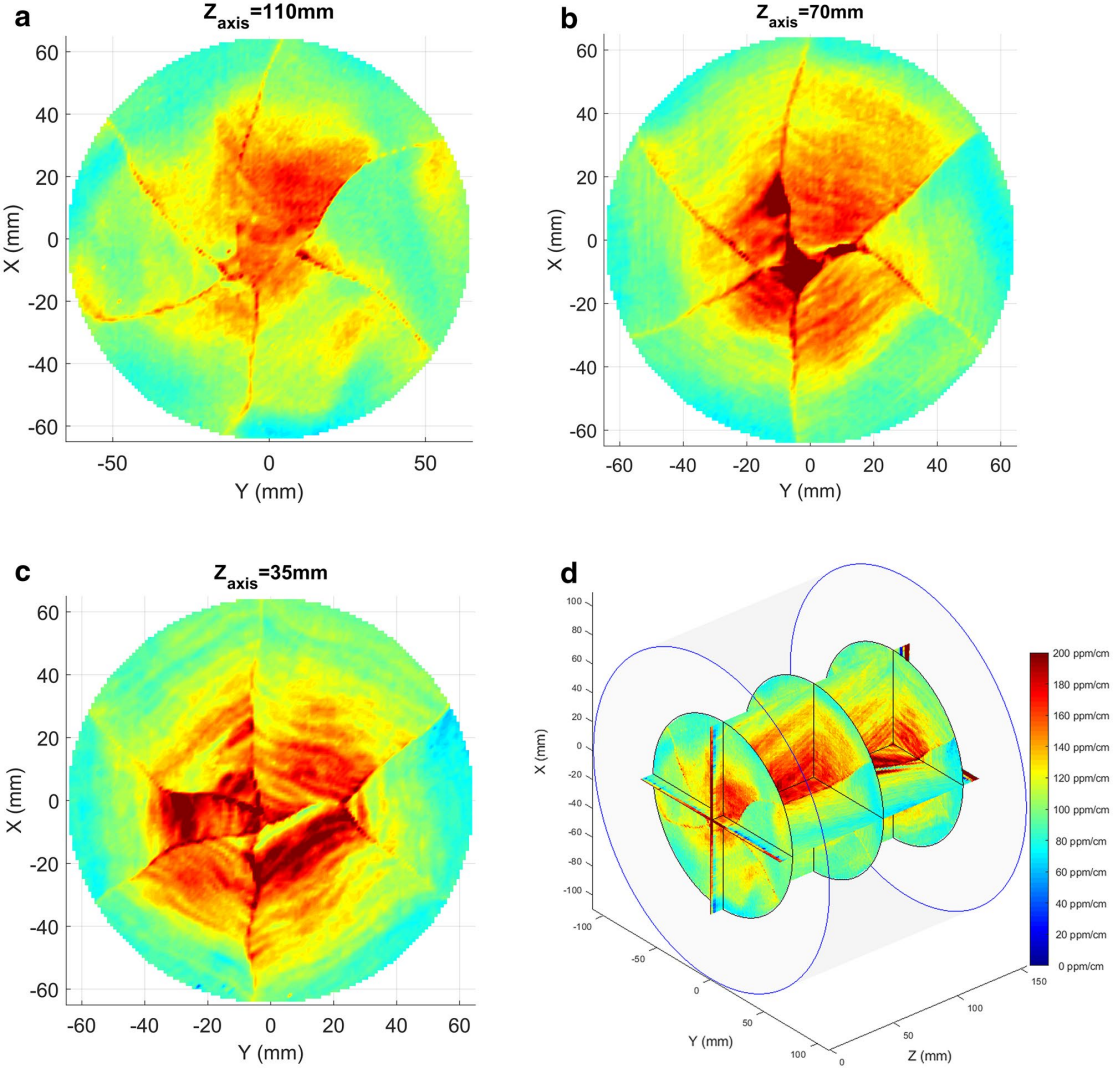
The circular absorption maps of the sapphire substrate at about 1 cm from the first surface **(a)**, in the middle of the sample **(b)**, and at about 1 cm from the second surface **(c)**. In the bottom-right figure **(d)** the 3D composition of the three circular maps together with the two rectangular one. A very good agreement in the spatial features of absorption can be observed.

The three XY maps of Sample A obtained at different positions along the growth directions are shown in Fig. 3. Here a remarkable star-like pattern with a sixfold symmetric shape is observed. From the maps, the pattern appears to develop inside the boule keeping the same orientation along the growth direction.

**Figure 4:**
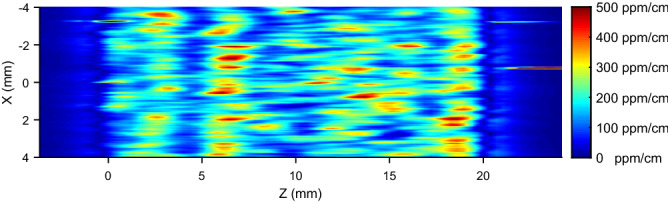
Rectangular map showing the XZ section of Sample B.

**Figure 5:**
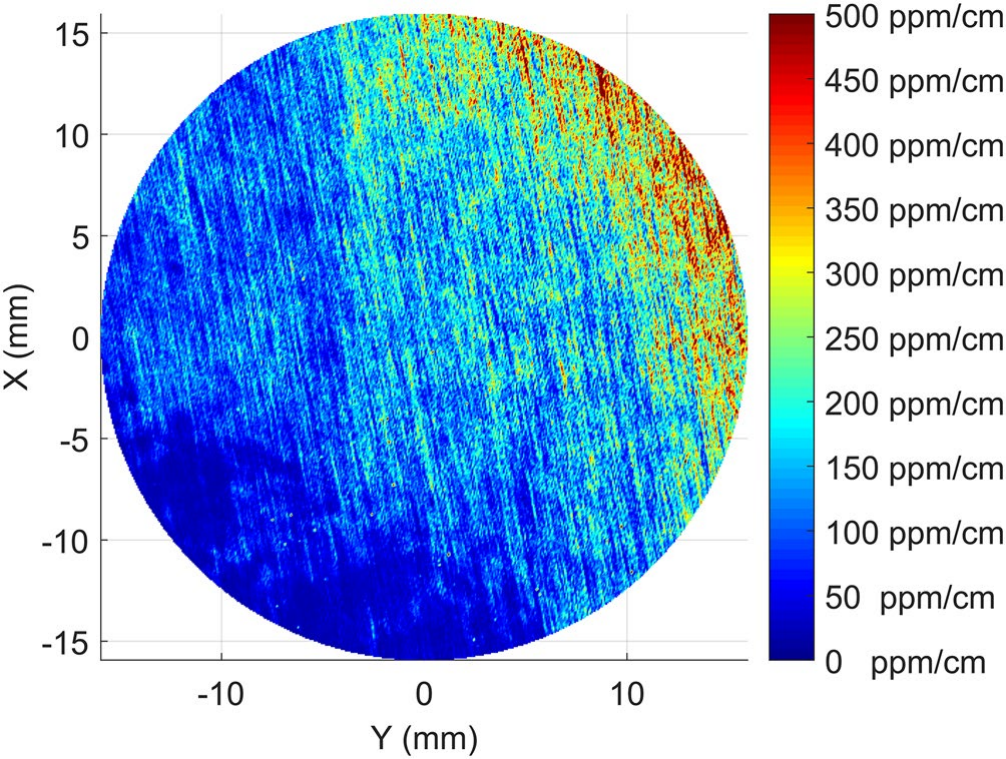
Circular absorption map of Sample B on the XY plane. A set of absorption features in the form of parallel stripes is roughly visible.

**Figure 6:**
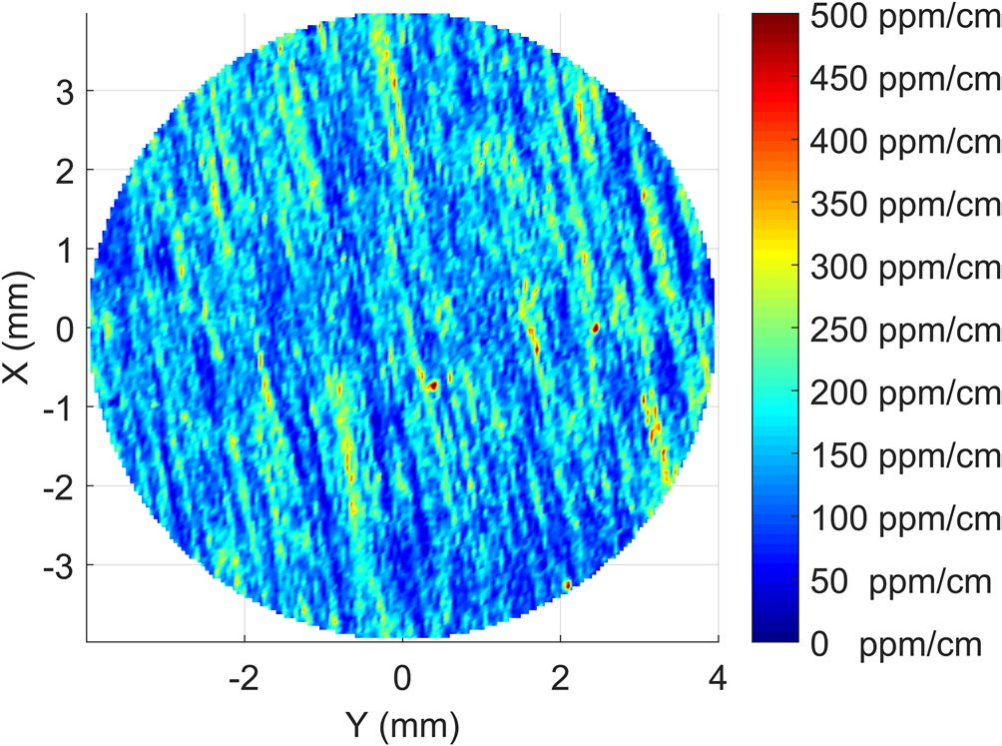
Zoom in the central region of Fig. 5 confirming the presence of absorption features in the form of parallel stripes.

The XZ map from Sample B is shown in Fig. 4. Contrarily to the former case, here the absorption map does not show any regular structure. Instead, the map on the XY plane in Fig. 5 shows a set of periodic stripes, confirmed also by the higher resolution map in Fig. 6. In this case, the absorption features appear to look quite different with respect to those observed in Sample A, in spite of the fact that they are produced with the same technique. The only difference is that the two samples are taken from different portions of two different boules.

## Discussion

Thanks to the three-dimensional space resolution of our system, we are able to uncover a non-homogeneous absorption distributed in both samples volume. Arguably, this can be attributed to the history of the samples either during its growth, either during its preparation. Those aspects will be detailed in the following.

The first notable observation in Fig. 2a,b is a strong absorption layer at both input and output surfaces. The thickness of those layers in our experimental maps is of few millimeters. Taking into account the spatial resolution of our system along the Z direction, it can be concluded that this area practically coincides with the sample surface and could be attributed e.g. to a non-optimal cleaning or residual contaminants from the polishing processes. However, the fact that in this specific condition the pump-probe interaction volume falls partly outside the sample makes unreliable the calibration procedure described in preceding sections. In the following we thus are not going to discuss quantitatively the data taken form these regions.

**Figure 7:**
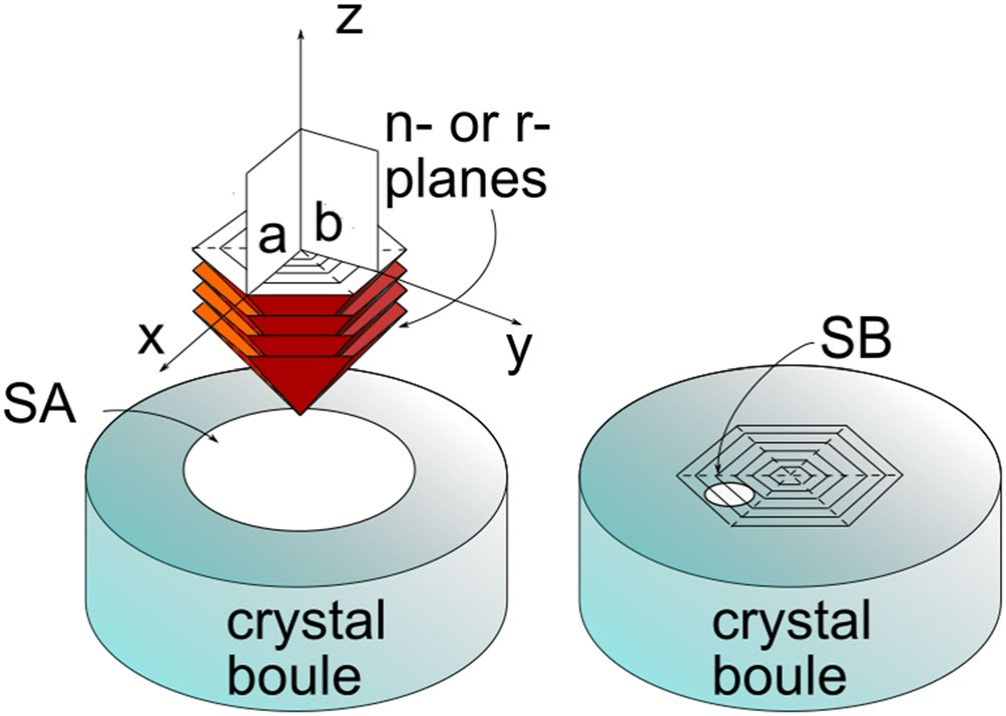
Scheme of the 3D absorption geometry as related to the growth facets associated to the n- and r-crystal plane families. The experimental maps of Fig. 2a,b corresponds to the planes labeled as “a” and “b” respectively. Sample A (SA) and sample B (SB) were carved from two different crystal boules from the positions shown in the scheme.

Second, the geometric shape of the absorption structure in Fig. 3a–c suggests that it may somehow be related to the growth history of the boule Sample A was taken from. By combining those maps with the views from Fig. 2a,b we infer that the absorption features are arranged inside the crystal volume along a set of pyramidal-like patterns that repeat themselves with a regular periodicity each 5–10 mm along the Z direction, see Fig. 7. These features point towards growth striations, a typical defect observed in sapphire crystals grown by different methods (see e.g. ref.^9^). Along the corners of those pyramidal structures the absorption appears to be higher and in particular close to the crystal axis, where all the pyramidal edges intersect . In the Kyropuolos growth, the liquid-solid interface inside the crucible has a pronounced convex shape that, differently from the Czochralski process, can expose strong growth planes. In particular, sapphire r-plane family with Miller indices $\{1,{\bar{1}},0,2\}$ and n-plane family $\{1,1,{\bar{2}},3\}$ form an acute angle of 32.4 and 29 degrees with respect to the Z direction and have a 3-fold and a 6-fold symmetry around it, respectively. This suggests that the pyramidal absorption stripes are related to those crystal plane families. They may be ascribed to compositional fluctuations occurring at the solid-liquid interface, also known as “growth striations”. In fact, the presence of growth striations periodically repeating along the growth planes is a well-known feature of melt-grown sapphire^9,10^ as facets constitute a preferential site for incorporation of impurities during the growth of sapphire in the Kyropoulos technique and any periodic instability of the growth parameters (melt temperature, melt convection, power fluctuations of the automatic growth system etc.) affects the segregation rate of impurities into the crystal. Among the others, in this case, the slow rotation of the crystal during the growth may be the origin of the periodicity.

Looking now at Sample B in Fig. 4 we can see that there is no evidence of any striation on the XZ plane. Periodic striations are however clearly visible in the XY plane (Fig. 5), yet with a smaller period of about 0.1–0.2  mm compared to the one of Sample A (see Fig. 6). To interpret this observation, it is necessary to recall that the spatial resolution of our setup in the XY plane is much higher than along the Z direction ($\simeq 0.05\, \hbox {mm}$ as compared to $\simeq 2\, \hbox {mm}$, respectively). We conclude that the striations are present also here, but with a smaller spatial period which cannot be observed in the XZ map due to the lower resolution of our setup along the Z direction. In our XY maps, the stripes are oriented at an angle of around 11 degrees with respect to the Y direction. By orienting the sample using X-ray data, it turns out that in the absorption measurement the sample was mounted so that the intersection of the crystallographic n-plane $\{1,1,{\bar{2}},3\}$ with the XY surface formed an angle of 8 degrees with respect to the Y direction which, taking into account the uncertainties due to sample mounting between the absorption and XRD setups, is in reasonable agreement with the orientation of the stripes. Those observations thus confirm that also in Sample B the observed absorption features coincide with growth striations probably due to impurities segregation along the crystallographic n-planes which are exposed to the melt due to the solid-liquid interface shape in the Kyropoulos technique.

It should be noted that the absence of any star-like pattern in the XY map of Sample B is coherent with our explanation. As this sample is obtained from an off-center region of the sample, the periodic striations do not display the form of a pyramid as in Sample A but rather a set of planes parallel to one of the pyramidal faces.

## Conclusions

We set up an experiment based on the PCI method to measure very low absorption sapphire substrates and we applied it to two samples obtained from two boules grown by the Kyropoulos technique along the *c*-axis. The first sample has a size compliant with the KAGRA design, the second one is a smaller one and is used for additional measurements. Note that, compared to other similar measurement methods previously applied to sapphire substrates for the KAGRA interferometer^11^, we are here able to provide a three-dimensional picture of the absorption features of our samples. This approach is here proven to be especially useful because it provides a significant amount of information helping to establish the possible origins of the increased absorption.

In the case explored in this work, the shape of the absorption structures suggests that they are related to growth striations, i.e. preferential incorporation of impurities along crystallographic growth planes of the n-family. This is a consequence of the characteristic convex shape of the solid-liquid interface in the Kyropoulos growth method and of the choice of the c-axis as pulling direction. The examined samples display the largest absorption (about 200 ppm/cm) located in the central region of the boule, which is highly undesirable in view of its application in gravitational wave detectors. This feature could cause deformations of the beam profile passing through the mirror, leading to the need of active correction systems, which are not foreseen at the moment in KAGRA.

The reasons for the striation emergence can be due either to a non-perfect control of the growth apparatus, or can be due to more “fundamental” reasons. The former cause is connected with temperature oscillations in the melt caused by natural convection and asymmetry of the thermal field created by non-uniformity and inconstancy of temperature at the crystallization front. An improvement of the striae intensity could thus be achieved by a choice of optimal temperature fields, lowering of gradients, raising of their stability, application of a mixing of the melt, and decrease of the total concentration of impurities, e.g. by trying different crucibles^9^.

Fundamental reasons besides periodic striae formation are related to basic transport phenomena of the impurities at the growth front^9^ (diffusion mixing as opposed to convective transport), and again this is determined by the choice of the growth technique. For the Kyropoulos technique, where impurity diffusion at the melt-solid interface should be the dominant transport mechanism, the striation period should be much shorter than the one observed here^9^. We therefore believe that the observed features are mainly due to the growth protocol. A decrease in the absorption could be possible by acting on the growth parameters, as discussed above. Clearly, different absorption structures are expected for crystals grown with other pulling directions or with other methods, possibly providing samples with either lower absorption or higher homogeneity or both.

Reducing the absorption in sapphire is of crucial importance for KAGRA and for future gravitational wave detectors. We believe that this type of characterization may support crystal growers in tailoring suitable growth processes allowing further improvements of sapphire mirrors quality.
